# Kidney Injury Molecule-1 (KIM-1) in Renal Cell Carcinoma: Biological Foundations and Emerging Clinical Applications

**DOI:** 10.3390/curroncol33070378

**Published:** 2026-06-23

**Authors:** Jason King Talao, Rohann Correa, Lakshman Gunaratnam, Ricardo Fernandes

**Affiliations:** 1 Division of Medical Oncology, Department of Oncology, Schulich School of Medicine & Dentistry, Western University, London, ON N6A 5W9, Canada; jason.talao@lhsc.on.ca; 2 Verspeeten Family Cancer Centre, London Health Sciences Centre, London, ON N6A 5W9, Canada; rohann.correa@lhsc.on.ca; 3 Division of Radiation Oncology, Department of Oncology, Schulich School of Medicine & Dentistry, Western University, London, ON N6A 5W9, Canada; 4 Division of Nephrology, Department of Medicine, Schulich School of Medicine & Dentistry, Western University, London, ON N6A 5W9, Canada; lakshman.gunaratnam@lhsc.on.ca; 5 Matthew Mailing Centre for Translational Transplant Studies, London Health Sciences Centre Research Institute, London, ON N6A 5A5, Canada; 6 Cancer Research Laboratory Program, Lawson Health Research Institute, London, ON N6A 4L6, Canada; 7 Department of Oncology, London Health Sciences Centre, Western University, 800 Commissioners Road East, Room A3-940, London, ON N6A 5W9, Canada

**Keywords:** renal cell carcinoma, kidney cancer, KIM-1, HAVCR1, TIM-1, biomarker, circulating biomarker, precision oncology

## Abstract

Renal cell carcinoma (RCC) is a biologically heterogeneous malignancy characterized by variable clinical behavior and diverse molecular phenotypes. Although immune checkpoint inhibitors and targeted therapies have transformed the treatment landscape of advanced RCC, clinically validated biomarkers capable of improving risk stratification, therapeutic-decision making and disease monitoring remain lacking. Kidney injury molecule-1 (KIM-1), also known as hepatitis A virus cellular receptor-1 (HAVCR1) or T-cell immunoglobulin and mucin domain-containing protein-1 (TIM-1), has emerged as a biologically compelling investigational biomarker e because of its close relationship to proximal tubular epithelial injury and renal carcinogenesis. KIM-1 is a transmembrane glycoprotein minimally expressed in normal kidney tissue but markedly upregulated in dedifferentiated proximal tubular epithelial cells following injury, and in clear cell RCC, where its extracellular domain can be shed into plasma and urine. Beyond its role as a marker of tubular injury, KIM-1 participates in immune regulation, phagocytosis, inflammatory signaling and tissue remodeling, supporting its potential relevance to tumor biology. Clinical studies have demonstrated associations between elevated circulating KIM-1 levels and RCC diagnosis, recurrence risk, and survival outcomes, particularly in localized and postoperative disease settings. KIM-1 has additionally been investigated as a therapeutic target through antibody–drug conjugate approaches. Despite promising translational data, important limitations yet remain. Current evidence is predominantly prognostic rather than predictive, and substantial analytical and biological challenges continue to limit implementation. Assay standardization, clinically meaningful cutoffs, specimen selection, timing of sampling, and confounding by chronic kidney disease or nonmalignant renal injury remain incompletely resolved. Furthermore, evidence supporting incremental value beyond established clinicopathologic models remains limited. This review critically evaluates the biological rationale, analytical considerations and clinical evidence supporting KIM-1 in RCC. Particular emphasis is placed on distinguishing prognostic, predictive, pharmacodynamic, and therapeutic applications, as well as defining the evidentiary gaps that must be addressed before clinical implementation. Current evidence is derived predominantly from retrospective and exploratory analyses, and important limitations remain regarding assay standardization, biological specificity, chronic kidney disease-related confounding, and prospective validation. The review concludes with a summary of the evolving landscape of KIM-1-directed biomarker strategies in RCC, which may ultimately contribute to improved biologic risk stratification and biomarker-driven clinical investigation in RCC.

## Simple Summary

Renal cell carcinoma, the most common form of kidney cancer, can behave very differently from one patient to another, making it difficult to predict who is most likely to experience recurrence or benefit from treatment. Despite major advances in immunotherapy and targeted therapies, reliable blood or urine biomarkers are still lacking in routine clinical care. Kidney injury molecule-1 (KIM-1) is a protein released by injured kidney cells and has emerged as a promising biomarker because it is closely linked to the type of kidney cells from which many kidney cancers arise. Recent studies suggest that higher levels of KIM-1 may be associated with the presence of kidney cancer, increased risk of recurrence after surgery, and poorer outcomes. This review summarizes the current understanding of KIM-1 biology, clinical evidence, and therapeutic potential, while also discussing important limitations and challenges that must be addressed before KIM-1 can be incorporated into routine patient care.

## 1. Introduction

Renal cell carcinoma (RCC) represents one of the most biologically heterogeneous malignancies in genitourinary oncology, encompassing diverse histologic, genomic, metabolic and immune phenotypes, associated with variable clinical outcomes [1,2,3]. Although major advances in immune checkpoint inhibitor (ICI)-based therapy and vascular endothelial growth factor receptor (VEGFR)-targeted therapies have reshaped the management of advanced disease, clinically validated biomarkers that reliably guide early detection, postoperative risk stratification, adjuvant treatment selection, and longitudinal disease monitoring remain lacking.

Historically, prognostic assessment in RCC has relied predominantly on clinicopathologic frameworks such as tumor stage, grade, necrosis, performance status, and composite models including the Leibovich score, UCLA Integrated Staging System (UISS), and International Metastatic RCC Database Consortium (IMDC) criteria. Although these models remain clinically useful, they do not fully capture the molecular and biological heterogeneity that drives recurrence risk and therapeutic response.

Despite extensive investigation of circulating tumor DNA (ctDNA), transcriptomic classifiers, radiomic approaches, and immune signatures, no circulating biomarker has yet been fully incorporated into routine RCC management. This gap is particularly relevant in localized and perioperative RCC, where improved biomarkers could help distinguish patients with occult residual disease from those cured by surgery alone.

Kidney injury molecule-1 (KIM-1), also referred to as Hepatitis A Virus Cellular Receptor-1 (HAVCR1) or T-cell and Mucin Domain Containing Molecule-1 (TIM-1), has gained increasing attention in RCC research as a potential biomarker because of its strong biological association with proximal tubular epithelial injury and tumor development [4,5]. Initially developed as a sensitive marker of proximal tubular injury, KIM-1 is minimally expressed in normal renal tissue but markedly upregulated in injured, regenerating, or dedifferentiated proximal tubular cells [4,5]. Importantly, clear cell RCC, the most common subtype, originates from proximal tubular epithelium, providing strong biological rationale for aberrant KIM-1 expression in malignant tissue [6,7,8].

Unlike static genomic alterations, circulating KIM-1 may provide a dynamic protein-based measure of tumor-associated and kidney-injury biology. However, whether circulating KIM-1 reflects tumor burden, residual disease, nonmalignant renal injury, treatment-related renal toxicity, or a combination of these processes remains incompletely understood.

**Figure 1:**
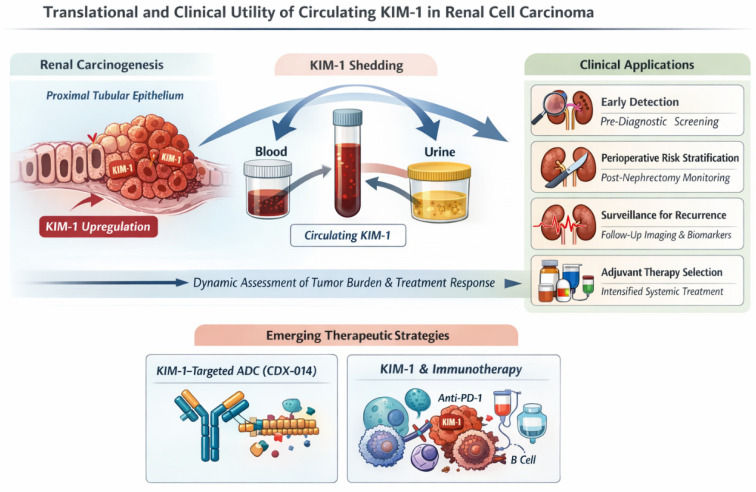
Proposed biological and clinical roles of KIM-1 in renal cell carcinoma. KIM-1 is minimally expressed in normal renal tissue but is upregulated in injured, dedifferentiated, and malignant proximal tubular epithelial cells. Its extracellular domain may be shed into plasma and urine, enabling non-invasive measurement. Arrows indicate proposed biological progression from proximal tubular injury and malignant transformation to KIM-1 upregulation, extracellular shedding into plasma and urine, and subsequent investigational biomarker and therapeutic applications across the RCC disease continuum. Potential clinical applications include early detection, postoperative risk stratification, longitudinal disease monitoring, and therapeutic targeting. These applications remain investigational and require prospective validation before routine clinical implementation. ADC: antibody drug conjugate; KIM-1: Kidney Injury Molecule-1; RCC, renal cell carcinoma. During the preparation of this manuscript, the authors used ChatGPT (GPT-5.3) to assist with formatting Figure 1. The prompts used were *“Create a scientific figure illustrating the biological role of KIM-1 in renal cell carcinoma, including KIM-1 expression, shedding into plasma and urine, and potential biomarker and therapeutic applications.”* and *“Refine the figure layout, labels, and visual presentation for clarity and suitability in a medical review article.”* The authors have reviewed and edited the output and take full responsibility for the content of this publication.

In recent years, translational and clinical studies have demonstrated associations between circulating KIM-1 levels and RCC diagnosis, recurrence risk, and survival outcomes [6,7,8,9,10]. Additionally, KIM-1 has been explored as a therapeutic target using antibody–drug conjugate (ADC) platforms [11]. However, important uncertainties remain regarding analytical validity, biological specificity, incremental clinical utility, and readiness for implementation. This narrative review synthesizes and critically evaluates the evolving role of KIM-1 in RCC, focused on: 1. the biological rationale linking KIM-1 to renal carcinogenesis; 2. distinctions between prognostic, predictive, pharmacodynamic, and therapeutic applications; 3. analytical and translational challenges limiting clinical implementation; and 4. the role of KIM-1 within the broader landscape of contemporary biomarker development in the immunotherapy era. The biological and potential clinical roles of KIM-1 across the RCC disease continuum are summarized in Figure 1.

## 2. Biological Foundation of KIM-1 in RCC

### 2.1. Structure and Physiological Function

KIM-1 is a type I transmembrane glycoprotein encoded by the HAVCR1 gene and expressed predominantly on proximal tubular epithelial cells following renal injury [4,12]. Structurally, KIM-1 contains an extracellular immunoglobulin-like domain, a mucin domain, a transmembrane segment, and a cytoplasmic tail involved in intracellular signaling. Under physiological conditions, KIM-1 expression in healthy kidney tissue is minimal or absent. However, expression is rapidly induced following ischemic, toxic, or inflammatory renal injury, where it contributes to epithelial dedifferentiation, phagocytosis of apoptotic debris, and tissue remodeling. Cleavage of its extracellular domain allows release into plasma and urine, enabling non-invasive measurement [4,12].

### 2.2. KIM-1 and Proximal Tubular Biology

The biological rationale for KIM-1 in RCC derives largely from its relationship to proximal tubular epithelial biology. Clear cell RCC, and to a lesser extent papillary RCC, originate from proximal tubular epithelium, where aberrant KIM-1 expression is biologically plausible and consistent with the cell of origin [6,7]. Although the strongest evidence supports KIM-1 expression in clear cell RCC, data regarding papillary RCC remain comparatively limited, while evidence in chromophobe RCC, collecting duct carcinoma, translocation-associated RCC, and other rare subtypes is sparse [7,8]. Consequently, most clinical conclusions regarding KIM-1 currently apply primarily to clear cell RCC and should not be generalized across all RCC histologies.

### 2.3. Immune Modulation and Tumor Biology

KIM-1 is increasingly recognized as more than a passive injury marker. Experimental studies suggest roles in immune modulation, phagocytosis of apoptotic debris, chronic inflammatory signaling and tissue remodeling [13,14,15]. Sustained KIM-1 expression has been linked to kidney fibrosis and chronic inflammatory states [16,17] that may contribute to carcinogenesis [8,17].

Additionally, KIM-1 signaling may intersect with immune regulation through effects on B-cell and T-cell function. Preclinical studies have suggested that TIM-1 signaling may influence antitumor immune responses, although the relevance of these pathways in human RCC remains incompletely understood. The biological significance of soluble KIM-1 also remains uncertain. While circulating KIM-1 likely reflects ongoing epithelial injury and tumor-associated shedding, it is unclear whether soluble KIM-1 acts solely as a biomarker or contributes functionally to tumor progression, immune modulation, or metastatic potential [8,17].

### 2.4. Tissue KIM-1 Versus Circulating KIM-1

Importantly, tissue KIM-1 expression and circulating soluble KIM-1 should not be considered biologically equivalent. Tissue KIM-1 reflects cellular expression within injured or malignant proximal tubular epithelial cells and may provide insight into local tumor biology. In contrast, circulating KIM-1 represents the shed extracellular domain and likely reflects a composite signal arising from tumor-associated shedding, renal injury, inflammation, and tissue remodeling. Consequently, conclusions regarding tissue expression cannot necessarily be extrapolated directly to circulating biomarker performance, and vice versa.

## 3. Analytical Validity and Translational Challenges

Although the biological rationale supporting KIM-1 in RCC is compelling, translation into routine clinical practice requires rigorous demonstration of analytical validity, clinical validity, and clinical utility. Importantly, these domains are distinct. Analytical validity refers to reliable and reproducible biomarker measurement; clinical validity concerns association with clinically meaningful outcomes; and clinical utility requires evidence that biomarker incorporation meaningfully improves patient management or outcomes [18,19].

Many biomarkers in oncology show promising associations with prognosis yet ultimately fail to achieve clinical implementation. For KIM-1 to achieve clinical utility in RCC, future studies must prove reproducibility across independent cohorts, incremental value beyond established clinicopathologic frameworks, and meaningful impact on clinical decision-making or patient outcomes [20].

### 3.1. Plasma Versus Urine Measurement

KIM-1 can be measured in both plasma and urine, but these matrices may reflect different biological processes Plasma KIM-1 may capture systemic shedding from tumor tissue, residual disease, or broader renal injury biology, and has therefore been evaluated in diagnostic and postoperative prognostic studies. In contrast, urine KIM-1 is more directly linked to tubular epithelial injury within the kidney and may be influenced by local renal injury, urinary concentration, baseline kidney function, proteinuria, and nonmalignant renal disease.

Early studies demonstrated that urinary KIM-1 was elevated in patients with RCC and could distinguish tumor-associated signal from adjacent normal tissue [6,7]. However, subsequent work has highlighted that KIM-1 is not cancer-specific and may also be elevated in acute kidney injury, chronic kidney disease, and other nonmalignant renal conditions [5,12]. Therefore, plasma and urine KIM-1 should not be treated as interchangeable biomarkers, and future studies should directly compare their diagnostic, prognostic, and longitudinal performance in RCC.

### 3.2. Assay Standardization

Assay heterogeneity represents a major barrier to implementation. Existing studies have utilized a variety of analytical including conventional enzyme-linked immunosorbent assays (ELISA), multiplex immunoassay approaches, and more recently high-throughput proteomic platforms [6,7,9]. Differences in assay design, antibody selection, calibration standards, lower limits of detection, and sample processing protocols may contribute to variability in reported KIM-1 concentrations across studies. Consequently, direct comparison between cohorts remains challenging.

Clinically meaningful thresholds remain undefined. Published studies have used different cutoff values and reporting strategies, and no universally accepted reference range currently exists for diagnostic, prognostic, or surveillance applications in RCC [17]. Standardization of assay methodology, calibration procedures, and reporting practices will be essential before KIM-1 can be considered for routine clinical implementation.

Moreover, timing of sampling may significantly influence interpretation. Preoperative, postoperative, longitudinal, and treatment-associated measurements may reflect distinct biological states and therefore require separate validation [9,10].

### 3.3. Confounding by Nonmalignant Renal Injury

One of the most important limitations of KIM-1 is its lack of cancer specificity. Elevated KIM-1 levels may occur in acute kidney injury, chronic kidney disease, diabetic nephropathy, ischemic injury, and inflammatory renal conditions [5,12]. This issue is particularly relevant because chronic kidney disease is common among patients with RCC. Chronic kidney disease and impaired renal function are common among patients with RCC owing to advanced age, hypertension, diabetes, and nephron loss following nephrectomy [1,21]. Consequently, elevations in circulating KIM-1 may reflect underlying renal injury rather than tumor-associated biology alone. Future studies should therefore incorporate adjustment for renal function, proteinuria, nephrectomy status, diabetes, hypertension and other competing renal comorbidities when evaluating KIM-1 performance.

### 3.4. Biological Interpretation of Circulating KIM-1

An additional unresolved question concerns the biological interpretation of circulating KIM-1. Elevated levels may reflect tumor burden, residual disease, inflammatory microenvironmental signaling, treatment-related renal injury, or combinations of these processes [9,10,17].

This complexity complicates interpretation of longitudinal changes and highlights the need for mechanistic studies linking circulating KIM-1 dynamics to tumor biology and treatment response.

### 3.5. Quality of Existing Evidence and Risk of Bias

A major limitation of the current KIM-1 literature is the predominance of retrospective, observational, and biomarker-exploratory studies. Many investigations involve relatively small patient cohorts, single-center datasets, post hoc analyses of clinical trials, or heterogeneous assay methodologies. Consequently, the available evidence remains vulnerable to selection bias, residual confounding, assay variability, and publication bias. Importantly, although prospective biomarker studies have been performed, KIM-1 has not yet been prospectively validated as a biomarker-integrated clinical decision-making tool, and its clinical utility remains unproven [9,10,17]. Therefore, current evidence should be interpreted as hypothesis-generating and supportive of further investigation rather than definitive evidence of clinical utility.

## 4. KIM-1 in the Context of Emerging Biomarkers in RCC

The development of circulating biomarkers in RCC has accelerated substantially over the past decade, particularly in the context of precision oncology and immunotherapy-based treatment strategies. However, no biomarker has yet been validated for routine treatment selection across localized or metastatic RCC. KIM-1 should therefore be considered within a broader biomarker landscape that includes circulating tumor DNA, transcriptomic signatures, radiomics, and immune-based biomarkers, each of which has important strengths and limitations.

### 4.1. Circulating Tumor DNA

ctDNA represents an attractive minimally invasive biomarker approach; however, RCC is characterized by relatively low tumor DNA shedding compared with other solid tumors. Detection sensitivity remains limited in localized disease, and ctDNA assays currently lack standardized clinical integration.

Both tumor-informed and tumor-uninformed ctDNA approaches are being evaluated in RCC [22,23]. Tumor-informed assays use sequencing of an individual patient’s tumor to identify specific variants that can subsequently be tracked in plasma, potentially improving specificity for minimal residual disease detection. In contrast, tumor-uninformed approaches do not require tumor tissue and may rely on predefined genomic, fragmentomic, methylation, or broader plasma-based signatures, but may be more vulnerable to lower sensitivity, background noise, and confounding from clonal hematopoiesis [22].

In RCC, ctDNA detection has been particularly challenging in localized disease because many renal tumors shed relatively low levels of tumor-derived DNA into circulation. As a result, a negative ctDNA result does not reliably exclude residual disease or future recurrence. Although ctDNA remains an attractive biomarker strategy, future studies will need to address challenges related to low tumor DNA shedding, assay sensitivity, and integration into perioperative management. Recent reviews have emphasized the importance of standardized approaches and prospective validation before routine clinical implementation [22].

KIM-1 may therefore offer complementary information as a protein-based biomarker linked to proximal tubular biology and tumor-associated shedding. However, unlike ctDNA, KIM-1 does not provide tumor-specific genomic information and may be confounded by nonmalignant renal injury [5,21]. Conversely, ctDNA possesses several important advantages over KIM-1, including high molecular specificity, direct assessment of tumor-derived genomic alterations, detection of resistance mechanisms, and potential applications in minimal residual disease assessment. Consequently, KIM-1 and ctDNA should be viewed as potentially complementary rather than competing biomarker approaches.

### 4.2. Transcriptomic and Molecular Signatures

Transcriptomic classifiers and molecular signatures have demonstrated prognostic and predictive potential in RCC, particularly in defining angiogenic and immune-inflamed phenotypes [24,25,26]. Nevertheless, these approaches typically require tissue acquisition and may not adequately capture temporal tumor evolution or intratumoral heterogeneity.

Several immunotherapy-era studies have evaluated angiogenesis, T-effector, myeloid inflammation, and immune-inflamed gene-expression signatures as candidate predictors of response to immune checkpoint inhibitor and VEGFR-directed combinations [23,26]. For example, biomarker analyses from trials such as IMmotion151 suggested that angiogenesis-high tumors may derive greater benefit from VEGFR-directed therapy, whereas T-effector or immune-inflamed signatures may enrich for response to immune checkpoint inhibition. However, the predictive performance of these signatures has been inconsistent across independent datasets. In JAVELIN Renal 101, for example, biomarker findings did not fully reproduce the same treatment-selection framework, underscoring the challenge of cross-trial validation [27].

Recent single-cell and spatial transcriptomic studies have highlighted substantial intratumoral metabolic and immune heterogeneity within clear cell RCC, further emphasizing the limitations of single-biopsy molecular assessment and supporting the need for complementary circulating biomarkers capable of longitudinal monitoring [28].

Accordingly, transcriptomic classifiers remain biologically informative but are not yet routinely used for treatment selection in RCC. Circulating protein biomarkers such as KIM-1 may complement these tissue-based approaches by providing a dynamic and repeatable measure of tumor-associated biology over time.

### 4.3. Radiomics and Imaging Biomarkers

Radiomic signatures and advanced imaging approaches have shown promise in RCC characterization and response prediction [24,29,30]. However, reproducibility, imaging standardization, and external validation remain major challenges.

Radiomic approaches extract quantitative imaging features such as tumor shape, texture, intensity, enhancement patterns, and spatial heterogeneity from computed tomography or magnetic resonance imaging. These features may provide non-invasive information about tumor phenotype, aggressiveness, and treatment response. However, radiomic biomarker development remains limited by variability in image acquisition protocols, segmentation methods, feature extraction pipelines, scanner differences, and statistical overfitting in small datasets.

External validation remains essential before radiomic biomarkers can be used clinically. Recent advances in radiomics and radiogenomics have further expanded interest in imaging-derived biomarkers in RCC. Radiogenomic approaches seek to link imaging phenotypes with underlying molecular and genomic characteristics, potentially enabling non-invasive assessment of tumor biology and therapeutic response [29,30]. However, substantial challenges remain regarding imaging standardization, feature reproducibility, external validation, and clinical implementation [24]. Future integrated biomarker strategies may combine radiomics, ctDNA, transcriptomics, and circulating protein biomarkers such as KIM-1 to improve precision risk stratification [24,28].

## 5. Clinical Applications Across Disease Settings

An important conceptual factor in KIM-1 research involves distinguishing among different biomarker applications. Prognostic, predictive, pharmacodynamic, and therapeutic biomarkers serve fundamentally different purposes and therefore require different evidentiary standards.

Much of the existing literature surrounding KIM-1 remains prognostic in nature. Therefore, caution is required when extrapolating prognostic association to treatment-selection utility or therapeutic implementation.

### 5.1. Early Detection and Diagnostic Applications

Importantly, distinct clinical applications of KIM-1 require different evidentiary standards. Current evidence remains strongest for prognostic association, while predictive and therapeutic applications remain investigational.

Several studies have demonstrated elevated circulating KIM-1 levels in patients with RCC compared with healthy controls [6,7,9,17]. Notably, pre-diagnostic analyses have suggested that elevated plasma KIM-1 may be detectable years prior to RCC diagnosis [9].

These findings raise the possibility that KIM-1 could contribute to future early detection strategies, particularly in high-risk populations. However, specificity remains a major limitation given the broad range of nonmalignant renal conditions associated with elevated KIM-1 [5,21].

At present, evidence is insufficient to support screening or diagnostic implementation.

### 5.2. Postoperative Risk Stratification

Postoperative risk stratification represents the most mature and clinically relevant area of investigation for circulating KIM-1 in RCC.

Despite curative-intent nephrectomy, recurrence remains common among patients with high-risk localized RCC. Current postoperative surveillance and adjuvant-treatment strategies rely primarily on clinicopathologic models that incompletely capture biological heterogeneity. Consequently, biomarkers capable of identifying minimal residual disease or occult micrometastatic burden represent a major unmet need.

Retrospective analyses from large prospective cohorts, including the ECOG-ACRIN E2805 (ASSURE) study, demonstrated that elevated postoperative plasma KIM-1 levels were independently associated with inferior disease-free survival (DFS) and overall survival (OS), even after adjustment for established clinicopathologic factors [10]. These findings support the hypothesis that circulating KIM-1 may reflect persistent residual disease biology not fully captured by conventional staging.

Nevertheless, several important limitations remain. Existing evidence derives largely from retrospective biomarker analyses rather than prospectively biomarker-integrated trials [10,17]. Standardized thresholds remain undefined, and performance across contemporary immunotherapy-era cohorts is uncertain. Moreover, demonstration of statistical association does not necessarily establish meaningful clinical utility [20,31].

Ultimately, demonstration of prognostic association alone is insufficient for clinical implementation. A clinically useful biomarker must improve discrimination, calibration, or risk reclassification beyond existing validated prognostic frameworks. At present, limited data exist evaluating whether KIM-1 significantly improves model performance metrics such as area under the receiver operating characteristic curve (AUC), concordance index (C-index), net reclassification improvement, or decision-curve analysis beyond established models including Leibovich, UISS, or IMDC [10,17]. This remains a critical evidence gap.

Accordingly, future studies should evaluate whether incorporation of KIM-1 meaningfully improves:

- Risk discrimination beyond validated prognostic frameworks;
- Risk reclassification across clinically relevant subgroups;
- Calibration of recurrence-risk estimates;
- Clinical decision-making regarding surveillance intensity or adjuvant therapy;
- Patient-centered outcomes and health-resource utilization.

### 5.3. Adjuvant Treatment Selection

The emergence of adjuvant immunotherapy has intensified interest in biomarkers that could help identify patients most likely to benefit from systemic treatment following nephrectomy [32]. Currently, no validated biomarker reliably predicts benefit from adjuvant immune checkpoint inhibition in RCC.

The strongest current clinical evidence supporting KIM-1 relates to postoperative prognostication following nephrectomy. In retrospective analyses of the ECOG-ACRIN E2805 (ASSURE) trial, elevated postoperative plasma KIM-1 levels were independently associated with inferior DFS and OS after adjustment for clinicopathologic variables. These findings suggest that circulating KIM-1 may reflect minimal residual disease or occult micrometastatic burden following surgery [10]. Nevertheless, further validation is required before integration into clinical decision-making.

Importantly, prognostic association alone does not establish clinical utility. Future studies must determine whether incorporation of KIM-1 meaningfully improves discrimination, calibration, or risk reclassification beyond established prognostic frameworks such as Leibovich, UCLA Integrated Staging System (UISS), or IMDC-related approaches.

Accordingly, KIM-1 should currently be viewed as a prognostic biomarker rather than a validated predictive biomarker for treatment selection [10,20].

### 5.4. Response Monitoring and Pharmacodynamic Applications

Serial circulating biomarkers may ultimately facilitate treatment monitoring, early response assessment, and relapse detection in advanced RCC. Longitudinal KIM-1 dynamics may potentially reflect changes in tumor burden or treatment-associated biological activity [9,10]. Nevertheless, interpretation is complicated by treatment-related renal injury, immune-related nephritis, VEGFR-associated renal toxicity, and baseline chronic kidney disease [5,21].

Prospective studies incorporating serial sampling alongside radiographic response assessment and translational correlatives are required before pharmacodynamic applications can be established [17,20].

## 6. Contemporary Immunotherapy-Era Relevance

The rapid evolution of RCC management in the immunotherapy era significantly alters the context in which biomarkers such as KIM-1 must be interpreted. ICI-based combinations are now widely used across metastatic and perioperative settings, including combinations involving nivolumab, pembrolizumab, ipilimumab, cabozantinib, lenvatinib, axitinib, and enfortumab vedotin-based approaches in related genitourinary malignancies. These therapies exert complex immunologic, inflammatory, and renal effects that may influence circulating biomarker dynamics.

Importantly, many historical biomarker studies were performed prior to routine integration of ICIs. Consequently, biomarker associations identified in tyrosine kinase inhibitor-era cohorts may not directly translate to modern treatment paradigms.

The perioperative immunotherapy setting is particularly relevant. Adjuvant pembrolizumab has demonstrated DFS and OS benefits in selected patients with high-risk localized RCC [32], intensifying the need for biomarkers capable of refining postoperative treatment selection. However, currently available evidence does not establish KIM-1 as a validated predictive biomarker for adjuvant immunotherapy benefit. Similarly, interpretation of serial KIM-1 measurements during systemic therapy may be complicated by treatment-associated nephrotoxicity, immune-mediated nephritis, VEGFR-related renal injury, or inflammatory signaling unrelated to direct antitumor activity. ICIs may further complicate interpretation of KIM-1 through induction of immune-mediated nephritis and inflammatory renal injury. Therefore, elevations in circulating KIM-1 during immunotherapy may not necessarily reflect tumor progression and could instead represent treatment-related renal toxicity. This issue remains largely unexplored and represents an important area for future investigation.

Future biomarker studies in RCC should therefore incorporate:

- Contemporary ICI-treated patient populations;
- Longitudinal sampling strategies;
- Integration with radiographic and molecular correlatives;
- Immune microenvironment characterization;
- Multi-analyte biomarker approaches combining ctDNA, transcriptomics, radiomics, and protein biomarkers.

In this evolving therapeutic landscape, KIM-1 may ultimately demonstrate utility as part of integrated biomarker frameworks rather than as an isolated biomarker.

## 7. Implications for Therapy and Drug Development

Key translational and therapeutic studies evaluating KIM-1 in RCC, including biomarker and drug-development investigations, are summarized in Table 1. The translational relevance of KIM-1 extends beyond biomarker development. Tumor-associated KIM-1 expression has motivated exploration of KIM-1-directed therapeutic strategies, particularly antibody–drug conjugates [8,11]. This therapeutic rationale is based on the surface expression of KIM-1/TIM-1 in a subset of RCC tumors, particularly clear cell RCC, and the ability of antibody–drug conjugates to deliver cytotoxic payloads to antigen-expressing tumor cells.

CDX-014 is the best-described KIM-1/TIM-1-directed therapeutic strategy in RCC. CDX-014 consists of a human anti-TIM-1 immunoglobulin G1 antibody linked to the cytotoxic payload monomethyl auristatin E. In a phase I study, patients with advanced or metastatic RCC, CDX-014 demonstrated manageable but clinically relevant toxicity and occasional durable responses in selected patients [33]. However, the study was limited by small sample size, a heavily pretreated population, heterogeneous TIM-1 expression, and the absence of a validated patient-selection strategy.

These findings suggest that KIM-1-directed therapy remains investigational. Future development will require more precise assessment of tumor KIM-1/TIM-1 expression, standardized companion diagnostic approaches, improved patient selection, and careful evaluation of on-target renal toxicity. In addition, the biological role of KIM-1 itself remains complex. Preclinical studies suggest that KIM-1 may participate in phagocytosis, immune regulation, inflammatory signaling, and tissue remodeling, raising the possibility that therapeutic targeting may have context-dependent effects beyond simple antigen delivery.

Beyond CDX-014, additional KIM-1/TIM-1-directed strategies may emerge as antibody–drug conjugate platforms continue to evolve. The growing interest in surface antigen-directed therapies in RCC is illustrated by the development of multiple therapeutic targets, including CD70, carbonic anhydrase IX, and KIM-1/TIM-1. Similar to CD70-targeted approaches, KIM-1-directed therapies may ultimately depend on reliable target expression assessment and appropriate patient selection [25]. At present, the available clinical evidence remains insufficient to establish KIM-1 as a validated therapeutic target in RCC.

## 8. Challenges and Future Directions

Future priorities should include: (1) assay standardization and cutoff definition; (2) prospective validation in contemporary immunotherapy-era cohorts; (3) rigorous adjustment for chronic kidney disease and nonmalignant renal injury; (4) evaluation of incremental value beyond established prognostic models; and (5) integration into multimodal biomarker frameworks incorporating ctDNA, transcriptomics, radiomics, and immune profiling.

Circulating KIM-1 levels may be influenced by non-malignant acute or chronic kidney injury), necessitating careful interpretation, particularly in patients with chronic kidney disease. Prospective validation studies are needed in contemporary immunotherapy-era cohorts. Assay standardization and clinically meaningful thresholds must be established. Future studies should rigorously evaluate incremental value beyond validated prognostic frameworks. Longitudinal biomarker-driven trials incorporating serial KIM-1 measurements may help define potential roles in minimal residual disease detection, relapse prediction, and treatment monitoring. Additionally, integrated biomarker strategies combining KIM-1 with ctDNA, transcriptomics, radiomics, and immune profiling may ultimately provide superior performance compared with single-analyte approaches.

Finally, continued investigation of KIM-1 biology may inform future therapeutic development [33], particularly in conjunction with next-generation antibody–drug conjugate platforms and immune-based therapeutic strategies.

## 9. Conclusions

KIM-1 represents a biologically compelling investigational biomarker in renal cell carcinoma. Current evidence supports associations between elevated circulating KIM-1 levels and adverse oncologic outcomes, while early translational studies additionally support exploration of KIM-1-directed therapeutic strategies. However, challenges remain before clinical implementation can be justified. Existing evidence is predominantly prognostic rather than predictive, and important uncertainties persist regarding assay standardization, clinically meaningful thresholds, biological specificity, and incremental utility beyond established clinicopathologic models. Accordingly, KIM-1 should currently be regarded as an investigational biomarker with strong biological rationale and early translational evidence but requiring prospective validation and rigorous clinical qualification before routine incorporation into RCC management.

## Figures and Tables

**Table 1:** Key Clinical and Translational Studies of KIM-1 in Renal Cell Carcinoma.

Study (Reference)	Year	Study Design/Setting	Population (No. of Patients)	Specimen (KIM-1 Assay)	Key Findings	Limitations	Clinical Relevance
Han et al. [6]	2005	Case–control diagnostic study	RCC (*n* = 75); Benign renal diseases (*n* = 68)	Tissue IHC; Urine ELISA	KIM-1 highly expressed in RCC tissue vs. benign tissues; Urinary KIM-1 significantly elevated in RCC	Single-center; relatively small cohort	First demonstration that KIM-1 is a tissue and urinary biomarker in RCC
Zhang et al. [7]	2014	Case–control diagnostic study	RCC (*n* = 84); Benign urologic disorders (*n* = 98)	Urine ELISA	Urinary KIM-1 levels higher in RCC than benign disorders; Good diagnostic performance (AUC 0.80)	No standardized cut-off; moderate sample size	Supports non-invasive urinary KIM-1 for RCC detection
Cuadros et al. [8]	2014	Tissue-based translational study	ccRCC (*n* = 218)	Tissue IHC, qRT-PCR	KIM-1 overexpression associated with IL-6/STAT3 activation in ccRCC; Linked to higher stage/grade and poorer outcomes	Retrospective; tissue-based only	Identified biological role of KIM-1 in ccRCC progression
Xu et al. [9]	2024	Multicenter prospective cohort (Preoperative)	RCC (*n* = 409); Benign renal masses (*n* = 205)	Plasma ELISA	Plasma KIM-1 distinguished RCC from benign masses (AUC 0.84); Higher levels associated with adverse pathological features and recurrence	External validation needed; median follow-up ~2 years	Strong evidence for plasma KIM-1 as a preoperative biomarker
Rini et al. [10]	2025	Prospective cohort (Adjuvant immunotherapy)	Localized RCC treated with adjuvant ICI (*n* = 263)	Plasma ELISA	Elevated circulating KIM-1 associated with shorter RFS and OS; Added prognostic value beyond clinicopathologic factors	Observational; predictive value not established	Suggests prognostic utility of KIM-1 in the immunotherapy era
He et al. [17]	2025	Systematic review and meta-analysis	27 studies; 3512 patients	Blood/Urine ELISA	Pooled results support diagnostic and prognostic performance of KIM-1; Significant heterogeneity across studies	Heterogeneity in assays, cut-offs and study design	Comprehensive synthesis confirming clinical potential
McGregor et al. [33]	2019	Phase I (First-in-human)	Advanced solid tumors including RCC (*n* = 34); RCC subset (*n* = 12)	Therapeutic targeting (CDX-014 ADC trial)	CDX-014 showed manageable safety and preliminary antitumor activity in RCC	Small sample size; early-phase study	Proof-of-concept for KIM-1-directed therapeutic strategies

KIM-1, kidney injury molecule-1; RCC, renal cell carcinoma; ccRCC, clear cell RCC; IHC, immunohistochemistry; ELISA, enzyme-linked immunosorbent assay; qRT-PCR, quantitative reverse transcription polymerase chain reaction; AUC, area under the curve; RFS, recurrence-free survival; OS, overall survival; ICI, immune checkpoint inhibitor; ADC, antibody–drug conjugate.

## Data Availability

This study is a review article and does not involve the generation or analysis of new data. All information is derived from previously published studies, which are cited within the manuscript.
